# Unpacking the effects of materialism on interpersonal relationships: A cognitive approach

**DOI:** 10.1111/bjso.12795

**Published:** 2024-08-26

**Authors:** Olaya Moldes

**Affiliations:** ^1^ Cardiff University Cardiff UK

**Keywords:** expectations, interpersonal relationships, materialism, media effects, relational conflict, relational satisfaction, social well‐being, standards

## Abstract

Materialism, or beliefs and values that link wealth and consumption to success and happiness, negatively affects interpersonal relationships. Prior work has typically explained these effects through the allocation of personal resources (such as time or money) within relationships, thus using a behavioural route. However, this research proposes an alternative cognitive pathway to understand the adverse effects of materialism on interpersonal relationships. Three studies (*N* = 1389) employing correlational and experimental methodologies showed that materialism leads to heightened expectations and standards for a significant other, which are associated with poorer interpersonal outcomes. Specifically, materialism heightens the ideal standards that one has for a close other around achievement (e.g., ambition) and positive image (e.g., attractiveness), which are linked to higher conflict and lower relational satisfaction. Therefore, this work contributes to deepening our understanding of how consumer‐oriented values shape social perceptions and negatively affect interpersonal dynamics. Practical applications include informing relationship counselling practices, developing educational interventions, and guiding marketers and media content producers towards messages that do not increase individuals' ideals and standards for themselves and others. Further research should explore other factors that might alter this mediation (e.g., mindfulness) and examine the short‐ and long‐term effects through longitudinal and interventional‐based research.

## BACKGROUND

Marketing communications often suggest that consumer products can enhance social connections. For example, the global campaign “*Share‐a‐Coke*” encourages individuals to foster or strengthen personal relationships through the shared consumption of carbonated beverages (Coca‐Cola, 2019; The Coca‐Cola Co., 2014). Similarly, campaigns like ‘*Cool Dad’* by Volkswagen (2017) have also highlighted how their product can improve the performance of the parental role and strengthen interpersonal relationships by increasing popularity and social status. However, commercial messages also promote materialism by implicitly communicating that consumer products are providers of happiness and signal one's personal worth and social success to others (Belk & Pollay, 1985; Shrum et al., 2022). Nevertheless, the endorsement of these ideas, which are key factors in the conceptualization of materialism (Dittmar et al., 2014; Richins & Dawson, 1992), has been found to have detrimental effects on developing and maintaining interpersonal relationships (e.g., Banerjee & Dittmar, 2008; Dunkeld et al., 2020; Hui & Tsang, 2017; Kashdan & Breen, 2007; Ward et al., 2020), therefore contradicting the social benefits initially promised by advertisements. It is worth noting that globally, the promotion and endorsement of materialism appear to be on the rise, as reflected in the consistent increase in marketing expenditure worldwide (Statista, 2023) and the continuous expansion of the international market for luxury and high‐end products (Bain & Company, 2023). Consequently, given the significant role of interpersonal relationships in one's health and well‐being (Cohen, 2004; OECD, 2020), research examining the underlying mechanisms through which materialism affects interpersonal relationships could help develop strategies to mitigate or counteract the adverse effects of materialism on well‐being in an increasingly consumer‐oriented society.

Most research examining the links between materialism and interpersonal relationships has explained these effects through motivational approaches (i.e., Self‐Determination Theory: Deci & Ryan, 2000). This perspective suggests that individuals who pursue extrinsic goals like wealth and social status often neglect their social connections because they invest their time in pursuing these goals. Experimental research supports this view, as studies have shown that exposure to materialistic advertisement messages reduces children's desire for peer contact (Goldberg & Gorn, 1978). Similarly, adults primed with the concept of ‘materialism’ were found to be less likely to seek social engagement (Bauer et al., 2012). However, the resource‐allocation approach overlooks the role of social cognition, which has consistently shown to play a significant role in interpersonal relationships (e.g., Baldwin, 1992; Campbell et al., 2001; Overall et al., 2006; Reis & Downey, 1999). This approach also fails to directly explain relational dynamics, which are key for understanding social bonds, particularly in the context of interpersonal conflict (e.g., Campbell et al., 2008; Roloff et al., 2006). Moreover, several findings in the materialism literature indicate that the temporal activation of this value affects people's relational dynamics. For example, children exposed to commercial advertisements were less likely to follow parental advice and were more prone to initiate a parent–child conflict (Goldberg & Gorn, 1978). Moreover, an observational study on couples showed that materialistic individuals were less able to connect with their partners (Hui & Tsang, 2017). This research showed that one's level of materialism did not impact the amount of self‐disclosure or emotional investment made in a communication exchange with a significant other. However, materialists were significantly less empathetic and understanding in their interactions with their partners. These results hint that cognitive processes might be playing a role in linking materialism with poor interpersonal relationships because the effects observed are not the result of a lack of time invested in the relationship, but they might be explained by the expectations that one holds for the other as a parent or romantic partner which could result in the negative relational dynamic observed. Indeed, prior studies have demonstrated that an endorsement of materialism widens the gap between one's actual and ideal self‐concepts, leading to lower self‐esteem and higher body dissatisfaction (Ashikali & Dittmar, 2012; Bauer et al., 2012; Richins, 1995; Teng et al., 2017). Therefore, building upon past research that has used cognitive approaches (e.g., Self‐Discrepancy Theory: Higgins, 1987) to explain the negative link between materialism and individual well‐being, this research proposes that materialism not only widens the gap between one's actual and ideal self but also affects the ideal standards one holds for close others, which in turn would result in poorer interpersonal relationships. Consequently, the present work will examine a social‐cognitive route as an alternative, yet complementary, explanation for the adverse effects found between the endorsement of materialism and interpersonal well‐being.

### Materialism and interpersonal relationships

Materialism extends beyond simply having or wanting money (Dittmar et al., 2014; Moldes & Ku, 2020; Shrum et al., 2013). Materialism is a psychological construct defined by beliefs and values that place money and possessions at the centre of one's life, link wealth and consumption with happiness, and assess individuals' worth based on the quantity and quality of their accumulated possessions (Dittmar et al., 2014; Richins & Dawson, 1992). Interestingly, research on materialism has often found a weak or non‐significant association between materialism and one's socioeconomic status (e.g., Li et al., 2018; Moldes et al., 2022; Wang et al., 2022) suggesting that one's wealth and the endorsement of materialism are not necessarily linked together. While money represents a tangible resource that can help an individual navigate their environment and expand or limit their choices, materialism serves as a mindset that shapes individuals' perceptions and directs their choices. Thus, ‘having money’ and ‘being materialistic’ are distinct factors that can have differential effects on one's social dynamics. This report focuses on the relationship between materialism and interpersonal well‐being because materialistic attitudes are internalized beliefs that can shift over time (Jaspers & Pieters, 2016; Moldes et al., 2022; Zawadzka et al., 2019), and thus they could potentially be modified when seeking to improve one's interpersonal bonds. Nevertheless, income and wealth are factors that might be more difficult to change as they often depend on external forces outside the control of individuals.

Interpersonal well‐being or relational health has been conceptualized as having positive contact with others and being actively involved in one's community (Bowling, 1991). Similarly, other researchers have measured one's satisfaction with their relationships and the quantity and quality of their social interactions (McDowell, 2006). Other authors have focused on one's perception of social integration (Keyes, 1998). The literature captures these differential approaches to interpersonal well‐being, as interpersonal relationships have been examined at a general social level (e.g., integration within a community or sense of belonging) and also at an individual level through dyadic interactions (e.g., satisfaction or conflict with one specific member of their social network).

When looking at the links between materialism and interpersonal relationships, studies looking at general social well‐being indicators suggest that materialism is associated with less positive relationships with others (Yoo et al., 2020) and higher loneliness (Loh et al., 2021; Manchiraju et al., 2021; Norris et al., 2012; Pieters, 2013). Moreover, studies looking at specific social roles have concluded that materialism is linked to lower relational satisfaction with one's parents (Allsop et al., 2021; Bae, 2016), romantic partners (Dean et al., 2007; Leavitt et al., 2019; LeBaron et al., 2017), and siblings (Kretschmer & Pike, 2010). Furthermore, materialism has also been linked to higher peer rejection (Banerjee & Dittmar, 2008) and parental conflict with adolescents (Ching & Wu, 2018). Therefore, the negative effects of materialism on interpersonal well‐being have been found at both the individual and the general level. The present research will examine interpersonal dynamics and relational satisfaction with a close other at an individual level to better unpack the specific cognitive mechanism underlying the associations between materialism and interpersonal relationships.

### Materialism and the self‐concept

The endorsement of materialism has been linked to lower self‐evaluations (Dittmar et al., 2014), larger self‐discrepancies between one's actual and ideal self (Dittmar, 2005), and higher self‐doubt (Chang & Arkin, 2002). Experimental research has also shown that exposure to advertisements showing luxury lifestyles and perfect bodies leads participants to focus more on their appearance and perceive higher self‐discrepancies between their current and ideal selves (Ashikali et al., 2017). Furthermore, researchers have also found that participants exposed to materialistic cues were less satisfied with themselves (Bauer et al., 2012) and attached higher importance to their appearance (Teng et al., 2017). These studies have often adopted a cognitive approach to explain the effects of materialism on one's self‐concept, frequently using Self‐Discrepancy Theory (SDT: Higgins, 1987) to support their findings (e.g., Dittmar, 2009; van den Berg et al., 2007). SDT suggests that we hold three different self‐concepts: (1) the *actual self*, or the self‐image and set of characteristics that one (and others) believe to have; (2) the *ideal self*, or the image and set of characteristics that one (and others) aspire to possess, and (3) the *ought self*, or the set of characteristics that one's (and others) belief that should have (Higgins, 1987). SDT postulates that having a significant gap between the *actual* and *ideal* image will lead to frustration and depressed emotions. However, to date, the cognitive mechanism has only been used to explain the adverse effects of materialism on self‐evaluations and has not been applied to interpersonal relationships. Consequently, this work aims to expand the materialistic value literature by applying a cognitive‐based approach to explain the negative effects of endorsing materialism on interpersonal relationships.

### Materialism and the concept of a close other

Cognitive theories such as SDT mention that we not only have a set of self‐concepts for ourselves but also for our significant others (Higgins, 1987). Moreover, self and identity researchers have also theorized that one self‐concept is intrinsically linked with the concept and knowledge that we hold for our close others (Andersen & Chen, 2002; James, 1984; Sedikides et al., 2011). Therefore, it is reasonable to expect that any effects on one's self‐concept could be extended to the concept we hold for a close other. Supporting this idea, experimental research has demonstrated that exposure to materialistic messages increases upward social comparison (Zhang & Zhang, 2016), suggesting that making materialism salient might make individuals more likely to engage in cognitive evaluation processes. Additionally, prior work looking at cognitive processes in interpersonal relationships has used SDT as a foundation to develop a model to be applied in romantic relationships. The Ideal Standard Model (Campbell et al., 2001; Fletcher et al., 1999) postulates that large discrepancies between the ideal and current perceptions of a romantic partner will lead to disheartenment, dejection, or discontent with the current partner. However, a limitation of this model is its failure to fully explain why individuals internalized varying ideal standards for others (Simpson et al., 2001). To address this gap, the present research proposes that materialism plays a role in shaping these internalized ideals. Consequently, this work also aims to extend the literature on interpersonal relationships by shedding some light on why ideal standards for others differ among individuals.

## THE PRESENT RESEARCH

This research aims to examine a cognitive‐based pathway to explain the adverse effects of materialism on one's interpersonal relationships. Drawing on the literature on materialism and interpersonal relationships (e.g., Banerjee & Dittmar, 2008; Dunkeld et al., 2020; Hui & Tsang, 2017; Kashdan & Breen, 2007; Ward et al., 2020), a negative association between materialism and indicators assessing the quality of one's interpersonal relationships would be expected.

Hypothesis 1 **(H1):** Higher levels of materialism will be associated with poorer interpersonal relationships.

Moreover, building on prior research that has found that the endorsement of this value leads to higher self‐ideals (e.g., Ashikali & Dittmar, 2012; Dittmar, 2005), it is hypothesized that materialism will also increase the ideal standards individuals hold for their close others. This extension is grounded on the notion that the concept that we have for a close other also forms part of one self‐concept (Andersen & Chen, 2002; James, 1984; Sedikides et al., 2011). Therefore, higher materialism would be expected to lead to higher ideal standards for others.

Hypothesis 2 **(H2):** Higher levels of materialism will lead to higher ideal standards for a close other.

**FIGURE 1:**
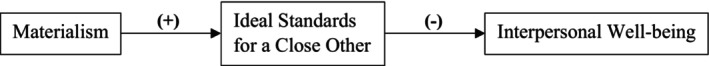
Conceptual model to be tested examining a cognitive pathway that links materialism with poorer interpersonal well‐being.

Finally, integrating cognitive theoretical perspectives looking at the perception of the self (Self‐Discrepancy Theory: Higgins, 1987) and close others (Ideal Standard Model: Fletcher et al., 1999), alongside prior findings on materialism and self‐evaluations (e.g., Ashikali & Dittmar, 2012; Dittmar, 2005), it is hypothesized that these heightened ideal standards for others will mediate the link between materialism and poor interpersonal relationships (see Figure 1). This suggests that the negative impact of materialism on interpersonal relationships will be partly explained by the heightened expectations that materialistic individuals hold for their close others.

Hypothesis 3 **(H3):** Higher ideal standards for a close other will mediate the link between materialism and the quality of interpersonal relationships.

The studies included in this report were approved by the Ethics Committee of the university where they were conducted.

## STUDY 1

The aim of Study 1 is to test H1, H2, and H3 with a correlational design by assessing the links between people's dispositional materialism, the current and ideal concept of a significant other, and their overall interpersonal conflict.

### Power analysis

#### Power for the correlational effects (H1 and H2)

For power analysis purposes, a small effect of *r* = −.18^1^ was forecasted between materialism and interpersonal conflict and between materialism and the discrepancies in other's actual and ideal concepts. Using G*Power, a sample size of 239 participants was estimated for a power of .80 (α = .05).

#### Power for the mediation effect (H3)

For power analysis purposes, the same effect size of *r* = −.18 used for the correlational analyses was forecasted for all the relationships between the variables in the mediation model. Using the open frame statistical program R with the package *pwr2ppl* (Aberson, 2019), a sample size of 380 was estimated for a power of 1 − β = .80 and α = .05.

### Methods and procedure

Four‐hundred participants were recruited through an online subject pool (Prolific) and took a short survey hosted by Qualtrics. Recruitment was limited to individuals 18 or older and residents of the United Kingdom. Participants first responded to the Materialistic Value Scale (MVS: Richins, 2004). Then, they were asked to think about a person close to them, describe their relationship (see Table A in Appendix S1 for a detailed account of the descriptions), and report their interpersonal conflict.^2^ Finally, they responded to two open questions in which they were asked to list positive and negative attributes of the person described before they completed the significant‐other‐discrepancies index and some demographic questions that included age, gender, and socio‐economic status.

### Sample

The final sample consisted of 394 participants,^3^ 73.9% of whom were females (*n* = 291), with ages ranging from 18 to 76 (*M*_Age_ = 39.27, SD_Age_ = 13.91), 49.2% (*n* = 194) indicated being full‐time employed, 20.3% were part‐time employed (*n* = 80), 19.3% marked other (*n* = 77) and described themselves as disabled, retired, homemaker, unemployed, or self‐employed, and 14% were students (*n* = 55; for further demographic characteristics see Table B in Appendix S1).

### Measures

#### Materialism

Materialism was measured with the 15‐item MVS, which uses a 5‐point scale ranging from 1 = ‘*strongly disagree*’ to 5 = ‘*strongly agree*’ (e.g., “*The things I own say a lot about how well I am doing in life*”; α = .85). Higher scores indicated higher endorsement of materialism.

#### Interpersonal conflict

Participants were asked to think about and name one person close to them, write their name, indicate their gender, describe their relationship nature (e.g., parent, romantic partner, etc.), and indicate their interpersonal conflict with them ‘*How often do you argue with this person?*’ in a 5‐point scale ranging from 1 = ‘*never*’ to 5 = ‘*almost always*’. This measure was taken from Mund and Neyer (2014).

#### Positive and negative attributes of the other

Participants were also asked to answer two open‐ended questions describing the positive and negative attributes of the other person. The display of the space to provide negative and positive attributes was randomized. A variable coding the number of positive attributes was created (e.g., for a response of “*Patient, Kind, Clever, Caring*”, a score of 4 was coded), and following the same principles, a variable with the number of negative attributes was also created (e.g., for a response of “*disloyal, narcissistic, bigoted*” a score of 3 was coded).

#### Significant‐other‐discrepancy index

The Self‐discrepancy Index (Ashikali & Dittmar, 2012; Dittmar, 2005) was adapted to evaluate the discrepancies perceived in a close other. Participants were asked to think about a close other and write three things they would change by completing the sentences *‘[Name of the person] is… But I would like them to be…*’ (e.g., ‘[Person described] is…*‘sometimes lazy’* but ‘I would like them to be *‘more ambitious*'), and rate how different was currently the person described from the ideal image that they have (from 1 = ‘*not very different*’ to 5 = ‘*very different’*). Following prior research procedures (Ashikali & Dittmar, 2012), the scores provided for the discrepancies were aggregated to create a significant‐other‐discrepancy index in which higher scores indicated higher discrepancies between the actual and ideal image the respondent holds for their close other.

### Results

#### H1 and H2: Materialism, perceived discrepancies in others and interpersonal conflict

Correlation analyses were performed to examine the relationship between the variables collected in the study (see Table 1). The results showed that materialism was significantly linked to the perceived discrepancies between the current and ideal image of the person described (*r* = .15) and conflict with that person (*r* = .19). Interestingly, the positive and negative attributes that the participants described from the person selected were not significantly linked to materialism (*p*s > .05). However, the higher number of negative attributes described was significantly linked with higher conflict (*r* = .19) and higher discrepancies (*r* = .32).

**TABLE 1:** Means, standard deviations, and correlations among the variables in Study 1 (*N* = 394).

	*M*	SD	2	3	4	5	6	7	8
1. MVS	2.78	0.66	0.19**	0.15**	−0.06	0.07	0.04	−0.28**	0.01
2. Interpersonal conflict	2.15	0.87	–	0.17**	−0.14**	0.19**	0.05	−0.11*	0.08
3. Others‐discrepancy index	6.58	3.11		–	−0.09	0.32**	0.00	−0.07	−0.17**
4. Positive attributes of the other	3.67	1.70			–	0.28**	0.13*	−0.07	−0.03
5. Negative attributes of the other	2.02	0.98				–	0.09	−0.04	−0.03
6. Gender	1.76	0.45					–	−0.10	0.03
7. Age	39.28	13.91						–	−0.06
8. SES	4.12	0.96							–

* *p* < .05 level (significance levels);

** *p* < .01, (2‐tailed).

#### Mediation (H3)

To test H3, a direct model in the open software R with the package *Lavaan* (Rosseel, 2012) was first conducted using the maximum likelihood estimation method and requesting 2000 bootstrapped 95% confidence intervals. The variable of conflict was introduced as a dependent variable, and dispositional materialism as an independent variable. The results showed a positive association between materialism and interpersonal conflict, β = .19, *p* < .001, 95% CI [.12, .38]. Then, a mediation model introducing the variable of discrepancy index as a mediator was conducted (see Figure A in Appendix S1). The results showed that materialism predicted higher conflict, β = .17, *p* = .001, 95% CI [.10, .35], and higher discrepancies in the other, β = .15, *p* = .003, 95% CI [.25, 1.17] and, that larger discrepancies in the other also predicted conflict, β = .15, *p* = .003, 95% CI [.01, .07]. Moreover, there was a total effect between materialism and conflict, β = .19, *p* < .001, 95% CI [.12, .38], as well as a significant mediation effect, β = .02, *p* = .035, 95% CI [.00, .06]. Alternative models controlling for demographic characteristics, including age, gender, and SES, produced equivalent results.

### Study 1 discussion

Study 1 shows that materialism is positively associated with interpersonal conflict, supporting H1. This finding is aligned with the effects found by prior correlational research between materialism and parental conflict in teenagers (Ching & Wu, 2018) but expands prior results by testing the effect in an adult population and looking at a wider range of relationships. Moreover, materialism was also positively linked with perceived higher discrepancies with a close other, supporting H2. Furthermore, the results also showed that higher discrepancies in the other partially mediated the relationship between materialism and interpersonal conflict, supporting H3. Interestingly, materialism was not found to be linked to the number of positive or negative attributes listed for the person described which suggests that higher endorsement of materialism does not influence the positive and negative characteristics identified in others but increases the expectations or idealized image that one holds for a close other.

## STUDY 2

Study 2 aimed to test the causal relationship between materialism and discrepancies for a close other using an experimental design in which a randomly selected group of participants was exposed to materialistic messages and compared with a control group.

### Power analysis

#### The mean difference between the manipulation and control group effect on the perceived discrepancies of the other (H2)

Based on the correlational effect found in Study 1 between materialism and self‐discrepancies (*r* = .15) an estimated effect of *d* = .30 was calculated for performing power analysis. Using G*Power, a sample size of 278 participants (139 per group) was estimated for a power of .80 (α = .05).

#### Power for the mediation effect (H3)

Based on the correlational effects found in Study 1 and using the open‐source statistical program R with the package *pwr2ppl* (Aberson, 2019), a sample size of 475 participants was estimated for a power of .80 (α = .05).

### Procedure

Five hundred and thirty participants were recruited from an online subject pool (Prolific) to complete a short questionnaire. Participation was limited to UK residents who were 18 years old or older. First, participants were randomly assigned to the materialism or the control condition, where they were asked to rate a series of pictures. Then, they completed a 6‐item scale about their materialistic orientations (α = .79) taken from Leyva (2018) before they were asked to think about a significant other and answer the same questions presented in Study 1.

### Sample

Of the final sample (*N* = 519),^4^ 70.3% were female (*n* = 365), ages ranged from 18 to 75 years old (*M*_Age_ = 39.13; SD_Age_ = 12.39), 59.3% reported being full‐time employed (*n* = 308), 19.5% were part‐time employed (*n* = 101), 6.2% were students (*n* = 32), and 18.7% selected other (*n* = 97) and described themselves as a homemaker, retired, self‐employed, or unemployed (for further demographic characteristics see Table B in Appendix S1).

### Conditions

#### Materialistic prime

Participants in the materialistic condition (*n* = 255) were presented with 12 images of high‐end fashion and luxurious lifestyles taken from magazine advertisements, following the procedures conducted by prior experimental research on materialism (Bauer et al., 2012; Leyva, 2018), and were asked to rate how much they liked each picture.

#### Control

Participants in the control condition (*n* = 264) saw 12 abstract graphic design images and were asked to rate how much they liked each picture.

### Results

#### Manipulation check

When looking at the differences between the materialistic prime and the control condition on their reported materialistic values, the results showed that there were no significant differences between the conditions, *t*(517) = .82, *p* = .411, with 2000 samples bootstrapped 95% CI [−.07, .19] suggesting that the exposure to materialistic images did not increase the participants' materialistic orientations. Moreover, the condition assigned (1 = materialism; 0 = control) was not significantly related to any of the variables in the study, suggesting that the manipulation did not cause any differences between the conditions (see Table 2). As a result, the data was analysed following Study 1 procedures.

**TABLE 2:** Means, standard deviations, and correlations among the variables in Study 2 (*N* = 519).

	*M*	SD	2	3	4	5	6	7
1. Condition	0.49	0.50	−0.04	0.04	0.04	0.07	0.01	−0.01
2. MVS	3.38	0.79	–	0.10*	0.12*	−0.07	−0.24**	0.08
3. Interpersonal conflict	2.20	0.91		–	0.22**	0.03	−0.05	0.02
4. Others‐discrepancy index	6.88	2.88			–	0.10*	−0.01	−0.03
5. Gender	1.71	0.47				–	−0.05	0.01
6. Age	39.13	12.34					–	−0.03
7. SES	4.07	0.92						–

* *p* < .05 level (significance levels);

** *p* < .01, (2‐tailed).

#### H1 and H2: Materialism, perceived discrepancies in others and interpersonal conflict

As in Study 1, correlation analyses were conducted between the variables collected in the study (see Table 2). Replicating the results of Study 1, dispositional materialism was significantly related to interpersonal conflict reported (*r* = .10), and the discrepancies perceived in the described significant other (*r* = .12). Moreover, the discrepancies perceived on the other were also significantly linked to conflict (*r* = .22).

#### Mediation (H3)

The same analyses and software used in Study 1 were used for Study 2 data. Therefore, an SEM direct model was conducted with the variable of conflict introduced as a dependent variable and dispositional materialism as an independent variable. The results revealed that materialism was a significant predictor of conflict, β = .10, *p* = .028, 95% CI [.01, .21]. Then, a mediation model was fit to the data, with the variable looking at the significant‐others discrepancy index as a mediator (see Figure B in Appendix S1). The results showed that materialism was no longer a significant predictor of conflict, β = .06, *p* = .205, 95% CI [−.04, .17]. However, they predicted the perceived discrepancies in the others, β = .12, *p* = .006, 95% CI [.13, .76], which predicted conflict, β = .21, *p* < .001, 95% CI [.04, .10]. Moreover, there was a marginally significant total effect between materialism and conflict, β = .10, *p* = .068, 95% CI [−.01, .20], and a significant mediation effect, β = .03, *p* = .017, 95% CI [.01, .06]. Equivalent results were obtained for alternative models controlling for individual differences in age, gender, SES, and the condition assigned.

### Study 2 discussion

The results from Study 2 replicated the findings from Study 1 using a different materialistic measure, showing that individuals higher in materialism also reported higher interpersonal conflict, supporting H1, and higher discrepancies in the concepts of a close other, supporting H2. Moreover, as in Study 1, the results showed a significant mediation of the perceived discrepancies in the other on the link between materialism and interpersonal conflict, supporting H3.

## STUDY 3

Study 3 aimed to test the causal link between materialism and the ideal standards we hold for a close other by improving the materialistic manipulation used in Study 2 and refining the measures collected to examine the others' ideals and the health of an interpersonal relationship. Study 3 also focused on romantic relationships because extended analyses of the prior data collected in Studies 1 and 2 indicated differences across scores among distinct social roles,^5^ and these types of relationships were also the most frequently mentioned in the previous studies (55% and 64% in Studies 1 and 2 respectively).

### Procedure

Following the power analyses conducted for Study 2, 476 participants were recruited from an online subject pool (Prolific) to complete a short questionnaire. Participation was limited to UK residents who were 18 or older and were currently in a romantic relationship. First, participants were randomly assigned to the materialism or the control condition. After the materialistic and control manipulations, all participants completed the 6‐item MVS scale (Richins, 2004). In the next section, participants were asked to rate a series of statements about their ideal partner. Finally, respondents were asked to think about their current partner and indicate their relationship satisfaction and interpersonal conflict.

### Sample

Of the final sample (*N* = 476), 50.4% were female (*n* = 240), ages ranged from 22 to 77 years old (*M*_Age_ = 44.74; SD_Age_ = 12.39), 60.3% reported being full‐time employed (*n* = 287), 19.5% were part‐time employed (*n* = 94), 1.7% were students (*n* = 8), and 19.5% selected other (*n* = 93) and described themselves as a homemaker, retired, self‐employed, or unemployed (for further demographic characteristics see Table B in Appendix S1).

### Conditions

#### Materialistic prime

Participants in the materialistic condition (*n* = 233) were asked to rate 11 statements taken from the 15‐item version of the MVS (Richins, 2004) after removing the items from the 6‐item short validated version of the scale. The scale presented ranged from 1 = ‘*neither agree nor disagree*’ to 5 = ‘*extremely agree*’, so participants were directed to indicate their agreement with the statements. Then, they were told that the system was computing their scores, and after, they were given feedback saying that their scores aligned with 88% of the respondents and that the results suggested that they believed that: (1) *Spending money can help in finding joy and happiness*; (2) *Money and possessions can reflect people's effort and are a sign of success*; (3) *Buying high‐end brands or enjoying luxurious experiences can give you a sense of achievement*; (4) *Acquiring wealth is important*. Then, they were asked to write a short essay‐type statement that justified their beliefs and illustrate these beliefs with one or more situations that made them come to those conclusions. Finally, they were shown four real luxury brand advertisements portraying couples and were asked to rate their likeability. The procedure to manipulate materialism adapted from prior research that had misleading scale items and provided bogus feedback to the participants (i.e., Barden & Petty, 2008; Moreno et al., 2021). A visual stimulus was also included following the procedures of prior materialistic manipulations (Leyva, 2018).

#### Control

Participants in the control condition (*n* = 243) rate the same 11 statements on the materialistic condition, but with a scale ranging from 1 = ‘*neither agree nor disagree*’ to 5 = ‘*extremely disagree*’, so they were directed to rate their disagreement with the statements. Then, they were presented with feedback saying that their scores aligned with 88% of the respondents and that they suggested that they believed that: (1) *Spending money is NOT a path to finding joy and happiness*; (2) *Money and possessions do NOT reflect one's worth or are a sign of success*; (3) *Buying high‐end brands or enjoying luxurious experiences does NOT give you a sense of achievement*; (4) *Acquiring wealth is NOT essential in life*. Then, as in the materialistic condition, they were asked to write a short essay to justify their beliefs. Finally, they were asked to rate how much they liked four abstract digital images.

### Measures

#### Materialistic Value Scale

After the manipulations were completed, all participants were asked to rate six statements from the 6‐item short version of the MVS (Richins, 2004) on a 7‐point scale ranging from 1 = ‘*strongly disagree*’ to 7 = ‘*strongly agree*’(α = .83).

#### Partner's ideals

To develop a scale, a content analysis was made with the data obtained from the open‐ended questions collected on Studies 1 and 2 in which participants needed to describe something they would change in their described closed other (*N* ≈ 2739 entries; see Table C in Appendix S1 for an overview of the themes identified). From the content analyses, 55 statements were initially generated from the changes mentioned more frequently. Then, these items were converted into an adjective (e.g., ‘*Better at planning and organising’* ➔ ‘*Organized*’; ‘*Respectful of boundaries and privacy*’ ➔ ‘*Respectful*’). Then, this list was compared with the 49 adjectives used by Fletcher et al. (1999) in their Ideal Partner Scale, and the exact duplicates (e.g., ‘*Supportive*’) were removed, obtaining a list of 63 items. The list developed through the content analysis from the data collected in Studies 1 and 2 was compared with the Ideal Partner Scale to understand if the same ideals were present in a UK sample collected more than two decades after their scale was developed. The comparison showed a high level of similarity, but new concepts around mental health management emerged (e.g., ‘*Resilient*’). Finally, to shorten the study materials, the adjectives close in meaning were merged to obtain a final list of 25 items (e.g., ‘*Confident*’ and ‘*Assertive*’ ➔ ‘*Confident*’; ‘*Considerate*’, ‘*Respectful*’, and ‘*Sensitive*’ ➔ ‘*Considerate*’; ‘*Financially secure*’ and ‘*Good job*’ ➔ ‘*Financially secure*’). In the final scale used, 19 items came from the original Partner Ideal Scale from Fletcher et al. (1999), and five were newly added. An exploratory factor analysis (principal components and Direct Oblim rotation) was conducted in SPSS. A five‐factor solution was suggested by the scree pot, and the variance explained (54.12%; see Table D in Appendix S1 for the factor loadings). The final five factors obtained were: (1) *Achievement* with 7 items (α = .81); (2) *Warmth and Reliability* with 6 items (α = .82); (3) *Independence and Openess* with 5 items (α = .79); (4) *Emotional Expression* with 4 items (α = .66); and (5) *Positive Image* with 3 items (α = .59). The correlations between the factors ranged from *r* = −0.36 to *r* = 0.19 suggesting some similarities, but also good independence between the factors.

#### Relationship satisfaction

Nine items^6^ from the Relational Satisfaction Scale (Røysamb et al., 2014) were used to measure perceived relational well‐being (α = .95).

#### Conflict

As in the previous studies, perceived conflict was measured using one item (‘*How often do you argue with [name of the person provided]?*’) that participants had to rate on a 7‐point scale ranging from 1 = ‘*Never*’ to 7 = ‘*Almost always’*.

### Results

#### Manipulation check

A *t*‐test was conducted to examine the differences between the conditions (1 = materialism; 0 = control) on materialistic values. The results showed that there were significant differences between the conditions, *t*(474) = −5.10, *p* < .001, Cohen's *d* = −.47, with bootstrapped 95% CI [.32, .72], showing that the participants in the materialistic manipulation (*M* = 3.94, SD = 1.21) had higher scores on the MVS Scale in comparison with the participants in the control condition (*M* = 3.42, SD = 1.01).

#### H1: Causal link between materialism and higher ideals for others

**FIGURE 2:**
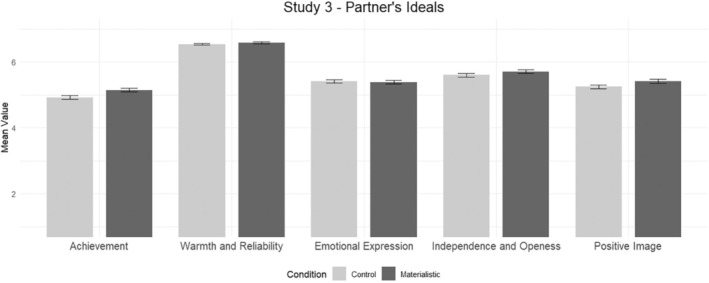
Bar chart displaying the means of the five factors identified in the Ideal Partner's Scale split by the manipulation (control vs. materialistic) in Study 3 (*N* = 476).

To test the link between materialism and the ideals hold for a close one, t‐tests were conducted with the condition assigned as the independent variable and the partner's ideals subscales as the dependent variables. The results show significant differences in achievement, *t*(474) = −2.80, *p* = .005, Cohen's *d* = .26, with bootstrapped 95% CI [−.07, −.38], and positive image, *t*(474) = −1.981, *p* = .048, Cohen's *d* = .18, with bootstrapped 95% CI [−.00, −.33]. Nevertheless, no other differences between the conditions were found for the other factors (*p*s > .05). These results show that manipulating materialism triggers a change in the ideal standards of a partner as participants in the materialistic condition rated their ideal standards for a partner higher in achievement (*M* = 5.14, SD = 0.87) and positive image (*M* = 5.41, SD = 0.94) than participants in the control condition (*M* = 4.92, SD = 0.86 and *M* = 5.24, SD = 0.89 respectively; see Figure 2).

Furthermore, correlational analyses (see Table 3) showed that the participant's chronic endorsement of materialism was significantly associated with achievement (*r* = .34), emotional expression (*r* = .11), and positive image (*r* = .22), suggesting that general levels of materialism are linked to higher ideal standards in a partner in those three domains.

**TABLE 3:** Means, standard deviations, and correlations among the variables in Study 3 (*N* = 476).

	*M*	SD	2	3	4	5	6	7	8	9	10	11	12
1. Condition	0.51	0.50	0.23**	0.13**	0.04	−0.02	0.07	0.09*	−0.07	−0.04	0.07	−0.05	−0.02
2. MVS	3.67	1.14	–	0.34**	0.02	0.11*	0.06	0.22**	−0.10*	0.07	0.00	−0.17**	0.04
3. Achievement	5.03	0.87		–	0.37**	0.47**	0.49**	0.55**	−0.12**	0.10*	0.24**	−.017**	0.12**
4. Warmth and reliability	6.55	0.52			–	0.45**	0.53**	0.32**	0.17**	−0.08	0.25**	−0.01	0.04
5. Emotional expression	5.40	0.86				–	0.48**	0.53**	0.11*	−0.03	−0.01	−0.12*	0.00
6. Independence and openness	5.65	0.84					–	0.51**	−0.01	0.03	0.12**	−0.09*	0.06
7. Positive Image	5.33	0.92						–	0.04	−0.06	−0.10*	−0.04	0.13**
8. Relational satisfaction	5.79	1.18							–	−0.58**	−0.14**	0.02	0.04
9. Conflict	2.87	1.15								–	0.17**	−0.04	−0.02
10. Gender	0.51	0.50									–	−0.08	−0.07
11. Age	44.74	12.39										–	0.05
12. SES	4.25	0.91											–

*Note*: Condition coded as 1 = ‘Materialistic condition’ and 0 = ‘Control’.

* *p* < .05 level (significance levels);

** *p* < .01, (2‐tailed).

#### H2: Materialism and relational well‐being

The causal link between materialism and relational well‐being was tested with a *t*‐test with the condition as the independent variable and relational satisfaction and conflict as independent variables. The results showed no significant differences between the groups (*p* values > .05). Nevertheless, correlational analyses showed that higher chronic materialism was significantly linked to lower relational satisfaction (*r* = −.10). These results suggest that a temporal manipulation of materialism might not necessarily cause a change in the relational satisfaction and conflict with a partner but that there is a correlational association between one's general materialism and their relational well‐being. These results suggest that one's relational satisfaction and conflict develop over time and might not be easily affected by temporal changes.

#### Mediation (H3)

**FIGURE 3:**
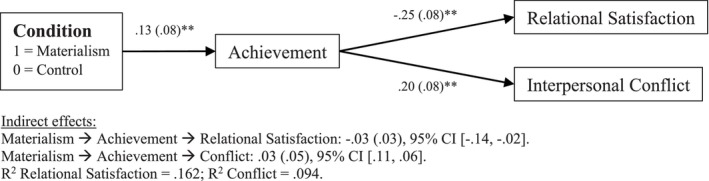
Mediation model testing H3 in Study 3 (*N* = 476). Only significant mediation effects have been displayed. Significance levels: **p < .01 (2‐tailed). See Table D in Appendix S1 for a full display of the results.

To test H3, a similar SEM model was performed with the same software used in Studies 1 and 2. However, in this case, the condition was added as the independent variable (coded as 1 = materialist condition and 0 = control condition). The five factors of *achievement*, *warmth and reliability*, *emotional expression*, *independence and openness*, and *positive image* were added as mediators, and relational satisfaction and interpersonal conflict were the dependent variables. The results showed that the condition only predicted achievement, β = .22, *p* = .005, 95% CI [.07, .38], and positive image. β = .17, *p* = .047, 95% CI [.00, .33], showing that participants in the materialistic condition had higher ideal standards on achievement and positive image. Moreover, the five factors looking at the partner's ideals were significant in predicting relational satisfaction and conflict, except emotional expression, which was not linked to conflict (see Table E in Appendix S1 for a full review of the results). Finally, the results also showed a significant indirect effect from materialism via achievement for both relational satisfaction and conflict (see Figure 3).

### Study 3 discussion

The results from Study 3 show a causal link between materialism and the standards one holds for a close other, confirming H2. However, it is worth noting that conflict and relational satisfaction are constructs that are formed over time through multiple and repeated interactions. Therefore, Study 3 was limited in confirming H1 with an experimental design. Looking at H3, the mediation models confirmed that the ideal standards of a partner in achievement and positive image mediated the links between materialism and relational well‐being.

## GENERAL DISCUSSION

The present research provides evidence that confirms a cognitive‐based pathway for understanding the effects of materialism on interpersonal relationships. Across three studies, it was observed that materialism influences the idealized standards individuals hold for their close others, which was subsequently linked to poorer interpersonal relationships. The mediation effects identified were consistent across three different data sets and were not affected by the demographic characteristics of the sample, showing the replicability and robustness of the proposed mechanism. The application of cognitive‐based approaches (e.g., Fletcher et al., 1999; Higgins, 1987) represents a step forward in understanding the links between materialism and interpersonal relationships, which to this date have been primarily attributed to behavioural patterns (i.e., Self‐Determination Theory: Ryan & Deci, 2000). This novel alternative explanation to the traditional behavioural‐based route should be considered complementary, as cognitive processes might precede or function alongside behavioural decision‐making mechanisms. Furthermore, this work also helped unpack the specific standards that materialism raises in others (Study 3), as achievement and positive image were directly affected when materialism was manipulated. Therefore, this work extends the current literature on materialism by testing an alternative route that explains the effects of this value on interpersonal relationships. Moreover, it also expands the literature looking at cognitive evaluations of others by identifying a specific factor (materialism) that can explain the variability across individuals on the internalized standards for close others. Overall, by further understanding the cognitive mechanisms that link materialism, close other perceptions, and interpersonal relationships, this research contributes to deepening our knowledge of how consumer‐oriented values, which are increasingly being promoted at a societal level, can shape interpersonal relationships and lays the ground for the development of interventions that could counteract the adverse effects of materialism on relational well‐being.

### Practical applications of the findings

First, the findings from these studies can be used to inform relationship and family counselling practices by helping therapists understand how materialism could influence relational dynamics. Therefore, counsellors could address materialistic‐related conflicts and work to develop strategies that would adjust expectations around achievements and a positive image of the other. Second, the findings from these studies could also be used to create educational programs or interventions that aim to bring awareness of the effects of endorsing materialism and consumer culture ideals by highlighting the impacts these beliefs have on the idealized expectations people hold for their close others and how they might also affect their relational dynamics. Third, the findings of this work could also help individuals to self‐reflect on their own materialism and how it might affect their interpersonal bonds so they can find ways to improve their relational dynamics. Finally, this research could also help raise awareness of the adverse effects of endorsing materialism and, thus, direct marketers and other media content producers towards creating materials that entertain or inform about the benefits of specific consumer products but that do not implicitly endorse materialism or distort individuals' ideals and standards for themselves and others.

### Limitations and further research

It is worth mentioning that the present report collected a one‐off and one‐sided measure of interpersonal well‐being. However, interpersonal relationships are dynamic processes (Barki & Hartwick, 2004; Coleman et al., 2012) that evolve over time. As a result, to deepen our current understanding of the role of cognitive processes in the link between the endorsement of materialism and the health of one's interpersonal relationships, further work should employ alternative methodologies, such as longitudinal research gathering data from two or more parties using diverse data collection methods (e.g., observational studies and/or relationship diaries) because qualitative data might better help us to deepen our understanding of interpersonal dynamics (Manning & Kunkel, 2014).

Second, the present research collected data from close others (Studies 1 and 2) and romantic relationships (Study 3). However, discrepancies in the nature of the relationship (e.g., romantic vs. friendship), as well as other factors such as power dynamics (e.g., parent–child) or financial dependency (e.g., homemaker partner), might influence the effects found. Therefore, future research should examine the effects presented in this report across different specific social roles, as these could shape the links between the factors.

Third, the mediation model presented in this report was replicated and showed robustness. However, other factors might influence the strength of this relationship. For example, further research should also examine other possible mediators, such as frustration or disappointment, because these negative emotions have been found to play an important role in negative interpersonal dynamics (Barki & Hartwick, 2004). Moreover, individual differences in mindfulness or emotional intelligence might influence the impact of materialism on the cognitive mechanism proposed, as hinted by recent research (Giacomantonio et al., 2022; Watson, 2019) and, thus, mitigate the adverse effects found on interpersonal relationships.

Fourth, materialism is socially learned through our early interactions with others (e.g., Richins, 2017; Shrum et al., 2022; Zawadzka et al., 2021) and through exposure to materialistic media messages (e.g., Dunkeld et al., 2020; Moldes et al., 2022; Shrum et al., 2005) and thus, it can be externally influenced by specific environments and messages. It is worth noting that it is unclear whether the manipulation used in Study 3 increased the materialistic orientations of the participants in the manipulation group or decreased the focus on materialism in the control group. Therefore, further interventional‐based research should examine whether reducing people's materialism can help improve one's interpersonal relationships or whether the effects observed in this report might be caused by continuous and consistent exposure to materialistic cues, which results in having distorted standards for others and thus, negative relational dynamics.

Finally, the present report collected data in the UK. However, cultural differences have been found in cognition and relationship evaluations (Endo et al., 2000; Ji et al., 2000). Therefore, to extend the generalizability of the findings, future research should be conducted in other countries, especially in regions where different cultural norms have been observed for interpersonal relationships.

## CONCLUSION

The findings from this report suggest that materialism heightens the ideal standards and expectations that one has for their significant others, particularly around achievement and positive image, which can translate into poorer interpersonal relationships. Therefore, this report found support for a cognitive‐based explanation for the negative effects found between materialism and interpersonal well‐being. Further research, especially longitudinal and intervention‐based, would be needed to understand how other factors might affect the processes found and determine the long versus short‐term effects of materialism on interpersonal dynamics.

## AUTHOR CONTRIBUTIONS

**Olaya Moldes:** Conceptualization; investigation; methodology; validation; visualization; writing – review and editing; writing – original draft; formal analysis; data curation; project administration.

## FUNDING INFORMATION

This research did not receive any specific grant from funding agencies in the public, commercial, or not‐for‐profit sectors.

## CONFLICT OF INTEREST STATEMENT

The author declares that there is no conflict of interest regarding the publication of this academic paper.

## Supporting information


Appendix S1.

## Data Availability

The data, the code used for the analyses, and the research materials are available at https://osf.io/3zpk8/.

## Footnotes

^1^
The estimated effect size of *r* = −.18 is the pooled effect obtained through meta‐analytical calculations using 120 effect sizes obtained from 53 published reports measuring materialism and interpersonal well‐being in Moldes et al. 2024.

^2^
Other measures, such as frequency of contact, closeness, and support, were collected, but no links with materialism were found, so they will not be reviewed further in this report.

^3^
In line with our hypothesis that the proposed effects would be observed in individuals close to the participant, we removed responses that contained a work colleague (*n* = 3) or other (*n* = 3) from the final sample as these responses represented distant others and were not aligned with our research focus.

^4^
Following the procedures from Study 1, responses that contained a work colleague (*n* = 5) or were described as other (*n* = 6) were removed from the final sample.

^5^
Significantly lower levels of conflict were found between friends and the rest of the social roles in both Study 1 and Study 2.

^6^
An item about children (‘*We agree on how children should be raised*’) was excluded to include couples without children.
