# Supplementary material for: Unpacking the effects of materialism on interpersonal relationships: A cognitive approach
Source: Br J Soc Psychol. 2024 Aug 26;64(2):e12795. doi: 10.1111/bjso.12795 (PMC11951399; doi:10.1111/bjso.12795)
Supplement: Supplementary file 1 — Appendix S1. [file BJSO-64-0-s001.docx]

## Supplementary Materials

**Chasing Wealth, Losing Connection: Unpacking the Interpersonal Strains of Materialism through Self-Discrepancy Theory**

## Table A.

*Summary of the descriptions of the close-other split by nature of the relationship in Study 1 and Study 2.*

|  |  | **Study 1** | **Study 2** |
| --- | --- | --- | --- |
| **Gender** | Female | 45.3% (*n* = 181) | 40.6% (*n* = 215) |
|  | Male | 54% (*n* = 216) | 59.1% (*n* = 313) |
|  | Other | 0.8% (*n* = 3) | 0.4% (*n* = 2) |
| **Social Role** | Romantic partner | 55% (*n* = 220) | 63.8% (*n* = 338) |
|  | Friend | 22.3% (*n* = 89) | 17% (*n* = 90) |
|  | Mother | 9% (*n* = 36) | 7.2% (*n* = 38) |
|  | Sibling | 5.3% (*n* = 21) | 4% (*n* = 21) |
|  | Child of their own | 3.5% (*n* = 14) | 3.4% (*n* = 18) |
|  | Father | 2.3% (*n* = 9) | 2.1% (*n* = 11) |
|  | Relative | 1.3% (*n* = 5) | 0.6% (*n* = 3) |
|  | Work colleague | 0.5% (*n* = 2) | 0.9% (*n* = 5) |
|  | Other | 1% (*n* = 6) | 1.1% (*n* = 6) |

## Table B.

*Summary of demographic characteristics of the samples used in Study 1 (N = 394), Study 2 (N = 519), and Study 3 (N = 476).*

|  | **Characteristics** | **Study 1** | **Study 2** | **Study 3** |
| --- | --- | --- | --- | --- |
| **Gender** | Females | 291 (73.9%) | 365 (70.3%) | 240 (50.4%) |
| **Age** | Mean age | 39.27 | 39.13 | 44.74 |
| **Race** | Whites | 339 (86%) | 453 (87.3%) | 429 (90.1%) |
|  | Asian | 23 (5.8%) | 24 (4.6%) | 19 (4%) |
|  | Blacks | 17 (4.3%) | 17 (3.3%) | 14 (2.9%) |
|  | Mixed | 15 (3.8%) | 23 (4.4%) | 14 (2.9%) |
| **Occupation** | Full-time employed | 194 (49.2%) | 308 (59.3%) | 287 (60.3%) |
|  | Part-time employed | 80 (20.3%) | 101 (19.5%) | 94 (19.7%) |
|  | Students | 55 (14%) | 32 (6.2%) | 8 (1.7%) |
|  | Other | 77 (19.3%) | 97 (18.7) | 93 (19.5%) |
| **Socio-Economic Status** | Average wealth | 169 (42.9%) | 242 (46.7%) | 196 (41.2%) |
|  | Above average wealth | 128 (32.5%) | 148 (28.6%) | 182 (38.2%) |
|  | Below average wealth | 59 (15%) | 84 (16.2%) | 55 (11.6%) |
|  | Moderately poor | 18 (4.6%) | 23 (4.4%) | 17 (3.6%) |
|  | Moderately rich | 13 (3.3%) | 16 (3.1%) | 16 (3.4%) |
|  | Very poor | 5 (1.3%) | 5 (1%) | 4 (0.8%) |
|  | Very rich | 1 (0.3%) | 0 | 1 (0.2%) |

## Table C.

*Summary of the themes found in a thematic analysis conducted on the three open-ended questions (‘I would like [name of the person described]…’; N* ≈ *2,739 responses) that participants responded as a part of the Self-Discrepancy Scale in Studies 1 (N = 394) and 2 (N = 519).*

| **Theme** | **Description** | **Examples** |
| --- | --- | --- |
| **Communication and Relationship Building** | - Listen attentively and avoid interrupting - Communicate openly about feelings and personal matters - Is considerate of others' opinions and feelings - Engage in meaningful conversations and show genuine interest - Understanding boundaries and respecting personal space | *‘… to respect my privacy’*  *‘… to be more open’*  *‘… to reach out to me more’*  *‘… to listen a bit more before she speaks’*  *‘… to be more vulnerable’*  *‘… to pay attention to the personal space of others’*  *‘… to be honest and discuss things when he is annoyed’* |
| **Self-Care and Well-Being** | - Effective emotional management - Stress and anxiety management - Self-care (appearance) - Self-care (fitness and diet) | *‘… to be more chilled’*  *‘… to handle stress better’*  *‘… to look after himself more’*  *‘… to react more calmly to stressful situations’*  *‘… to limit his snacking’*  *‘… to not smoke’*  *‘… to be more fitness-focused and sports-minded’*  *‘… to have a better-balanced diet’* |
| **Self-Awareness and Personal Growth** | - Recognising personal flaws and working on them - Seeking feedback and being willing to change - Being self-aware and understanding one's impact on others | *‘… to be able to see things from someone else's point of view’*  *‘… to see both sides of a story’*  *‘… to be less judgemental’*  *‘… to be more open-minded’*  *‘… to understand that she isn't right all the time and doesn't know everything’* |
| **Mindset and Attitude** | - Being more positive and optimistic - Embracing change and being adaptable - Take initiative and be proactive - Self-reliant - Ambitious | *‘… to be more confident’*  *‘… to think positively’*  *‘… to be more proactive’*  *‘… to be more patient’*  *‘… to be more independent’*  *‘… to be true to himself’*  *‘… to be more adventurous’*  *‘…to get a more successful job’*  *‘… to be assertive’* |
| **Time Management, Organization Skills, and Budgeting** | - Balancing work and personal life - Time management skills - Saving and spending money wisely | *‘… to be tidier’*  *‘… to properly time manage’*  *‘… to help more with housework’*  *‘… to spend more time with family’*  *‘… to be more financially aware’*  *‘… to be on time’* |

## Table D.

*Factor loadings from the 25 items measuring Partner’s Ideals.*

|  | **1**  **Achievement** | **2**  **Warmth and Reliability** | **3**  **Emotional Expression** | **4**  **Independence and Openness** | **5**  **Positive Image** |
| --- | --- | --- | --- | --- | --- |
| Successful | **0.817** | 0.100 | 0.075 | 0.030 | -0.023 |
| Financially secure | **0.758** | -0.093 | -0.132 | 0.119 | 0.123 |
| Ambitious | **0.679** | 0.133 | 0.218 | -0.126 | -0.312 |
| Organised | **0.601** | -0.005 | -0.004 | -0.074 | 0.113 |
| Hard-working | **0.591** | -0.230 | 0.041 | -0.147 | -0.139 |
| Well-groomed | **0.537** | -0.098 | 0.181 | 0.024 | 0.298 |
| Stable | **0.452** | -0.368 | -0.315 | -0.106 | 0.182 |
| Trustworthy | -0.033 | **-0.810** | -0.040 | 0.102 | 0.120 |
| Supportive | 0.010 | **-0.729** | 0.187 | -0.002 | -0.082 |
| Kind | -0.064 | **-0.701** | 0.101 | -0.009 | 0.123 |
| Considerate | 0.015 | **-0.684** | 0.070 | -0.074 | -0.068 |
| Reliable | 0.181 | **-0.656** | -0.119 | -0.126 | -0.075 |
| Understanding | 0.029 | **-0.564** | 0.079 | -0.266 | -0.317 |
| Affectionate | -0.120 | -0.402 | **0.692** | 0.107 | 0.131 |
| Romantic | 0.112 | -0.096 | **0.689** | -0.019 | 0.053 |
| Adventurous | 0.188 | 0.094 | **0.393** | -0.369 | -0.013 |
| Generous | 0.224 | -0.093 | **0.366** | -0.241 | 0.024 |
| Open-minded | -0.216 | -0.054 | 0.164 | **-0.756** | -0.053 |
| Independent | 0.100 | 0.043 | -0.145 | **-0.695** | 0.126 |
| Self-aware | 0.121 | -0.139 | 0.077 | **-0.577** | -0.185 |
| Resilient | 0.169 | -0.202 | -0.166 | **-0.555** | 0.054 |
| Easy-going | -0.105 | -0.148 | -0.050 | **-0.443** | 0.392 |
| Confident | 0.241 | -0.031 | 0.217 | -0.384 | **0.175** |
| Attractive | 0.240 | 0.068 | 0.360 | -0.017 | **0.544** |
| Intelligent | 0.093 | 0.037 | 0.088 | -0.373 | **0.449** |

## Table E.

*Unstandardised and standardised estimates, standard errors, significance levels, and bootstrapped 95% confidence intervals for the mediation model in Study 3 (N = 476) predicting Relational Satisfaction (RS) and Conflict (C) by the Condition assigned and the Partner’s Ideal Standards.*

| **Path** | **Unstandardised estimates** | **Standardised estimates** | **Standard Error** | ***p*** | **Bootstrapped 95% CI** |
| --- | --- | --- | --- | --- | --- |
| Condition 🡪 Achievement | 0.223 | 0.223 | 0.079 | 0.005 | [0.067, 0.378] |
| Condition 🡪 Warmth and Reliability | 0.038 | 0.038 | 0.048 | 0.424 | [-0.055, 0.131] |
| Condition 🡪 Emotional Expression | -0.028 | -0.028 | 0.079 | 0.718 | [-0.182, 0.126] |
| Condition 🡪 Independence and Openness | 0.111 | 0.111 | 0.076 | 0.147 | [-0.039, 0.260] |
| Condition 🡪 Positive Image | 0.166 | 0.166 | 0.084 | 0.047 | [0.002, 0.331] |
| Condition 🡪 RS | -0.097 | -0.097 | 0.105 | 0.353 | [-0.302, 0.108] |
| Condition 🡪 C | -0.121 | -0.121 | 0.104 | 0.246 | [-0.325, 0.083] |
| Achievement 🡪 RS | -0.355 | -0.355 | 0.060 | 0.000 | [-0.472, -0.238] |
| Warmth and Reliability 🡪 RS | 0.562 | 0.562 | 0.099 | 0.000 | [0.367, 0.756] |
| Emotional Expression 🡪 RS | 0.175 | 0.175 | 0.060 | 0.004 | [0.057, 0.293] |
| Independence and Openness 🡪 RS | -0.176 | -0.176 | 0.062 | 0.004 | [-0.297, -0.055] |
| Positive Image 🡪 RS | 0.129 | 0.129 | 0.056 | 0.022 | [0.019, 0.240] |
| Achievement 🡪 C | 0.267 | 0.267 | 0.059 | 0.000 | [0.151, 0.383] |
| Warmth and Reliability 🡪 C | -0.322 | -0.322 | 0.099 | 0.001 | [-0.515, -0.128] |
| Emotional Expression 🡪 C | -0.031 | -0.031 | 0.060 | 0.609 | [-0.148, 0.087] |
| Independence and Openness 🡪 C | 0.141 | 0.141 | 0.062 | 0.022 | [0.020, 0.262] |
| Positive Image 🡪 C | -0.196 | -0.196 | 0.056 | 0.000 | [-0.306, -0.086] |
| **Indirect effects:** |  |  |  |  |  |
| Condition 🡪 Achievement 🡪 RS | -0.079 | -0.032 | 0.031 | 0.011 | [-0.140, -0.018] |
| Condition 🡪 Warmth and Reliability 🡪 RS | 0.021 | 0.009 | 0.027 | 0.429 | [-0.032, 0.074] |
| Condition 🡪 Emotional Expression 🡪 RS | -0.005 | -0.002 | 0.014 | 0.720 | [-0.005, 0.007] |
| Condition 🡪 Independence and Openness 🡪 RS | -0.019 | -0.008 | 0.015 | 0.197 | [-0.049, 0.010] |
| Condition 🡪 Positive Image 🡪 RS | 0.022 | 0.009 | 0.014 | 0.133 | [-0.007, 0.050] |
| Condition 🡪 Achievement 🡪 C | 0.059 | 0.025 | 0.025 | 0.017 | [0.011, 0.108] |
| Condition 🡪 Warmth and Reliability 🡪 C | -0.012 | -0.005 | 0.016 | 0.437 | [-0.043, 0.019] |
| Condition 🡪 Emotional Expression 🡪 C | 0.001 | 0.000 | 0.003 | 0.768 | [-0.005, 0.007] |
| Condition 🡪 Independence and Openness 🡪 C | 0.016 | 0.007 | 0.013 | 0.221 | [-0.009, 0.041] |
| Condition 🡪 Positive Image 🡪 C | -0.033 | -0.014 | 0.019 | 0.084 | [-0.070, 0.004] |

## Table F.

*Unstandardised and standardised estimates, standard errors, significance levels, and bootstrapped 95% confidence intervals for the mediation model in Study 3 (N = 476) predicting Relational Satisfaction (RS) and Conflict (C) by MVS, the Partner’s Ideal Standards, and controlling for the condition assigned.*

| **Path** | **Unstandardised estimates** | **Standardised estimates** | **Standard Error** | ***p*** | **Bootstrapped 95% CI** |
| --- | --- | --- | --- | --- | --- |
| MVS 🡪 Success | 0.253 | 0.253 | 0.034 | 0.000 | [0.187, 0.320] |
| Condition 🡪 Achievement | 0.091 | 0.091 | 0.077 | 0.238 | [-0.060, 0.242] |
| MVS 🡪 Warmth and Reliability | 0.006 | 0.006 | 0.021 | 0.784 | [-0.036, 0.048] |
| Condition 🡪 Warmth and Reliability | 0.035 | 0.035 | 0.049 | 0.474 | [-0.061, 0.131] |
| MVS 🡪 Emotional Expression | 0.086 | 0.086 | 0.035 | 0.014 | [0.017, 0.155] |
| Condition 🡪 Emotional Expression | -0.073 | -0.073 | 0.080 | 0.360 | [-0.231, 0.084] |
| MVS🡪 Independence and Openness | 0.033 | 0.033 | 0.034 | 0.338 | [-0.034, 0.100] |
| Condition 🡪 Independence and Openness | 0.094 | 0.094 | 0.078 | 0.233 | [-0.060, 0.247] |
| MVS 🡪 Positive Image | 0.165 | 0.165 | 0.037 | 0.000 | [0.093, 0.238] |
| Condition 🡪 Positive Image | 0.080 | 0.080 | 0.084 | 0.340 | [-0.085, 0.246] |
| Achievement 🡪 RS | -0.333 | -0.333 | 0.063 | 0.000 | [-0.457, -0.210] |
| Warmth and Reliability 🡪 RS | 0.554 | 0.554 | 0.099 | 0.000 | [0.360, 0.749] |
| Emotional Expression 🡪 RS | 0.174 | 0.174 | 0.060 | 0.004 | [0.055, 0.292] |
| Independence and Openness 🡪 RS | -0.185 | -0.185 | 0.062 | 0.003 | [-0.306, -0.063] |
| Positive Image 🡪 RS | 0.135 | 0.135 | 0.057 | 0.019 | [0.022, 0.247] |
| MVS 🡪 RS | -0.044 | -0.044 | 0.050 | 0.377 | [-0.143, 0.054] |
| Condition 🡪 RS | -0.079 | -0.079 | 0.106 | 0.459 | [-0.287, 0.130] |
| Achievement 🡪 C | 0.237 | 0.237 | 0.062 | 0.000 | [0.115, 0.360] |
| Warmth and Reliability 🡪 C | -0.312 | -0.312 | 0.099 | 0.002 | [-0.505, -0.118] |
| Emotional Expression 🡪 C | -0.029 | -0.029 | 0.060 | 0.629 | [-0.147, 0.089] |
| Independence and Openness 🡪 C | 0.152 | 0.152 | 0.061 | 0.013 | [0.032, 0.273] |
| Positive Image 🡪 C | -0.204 | -0.204 | 0.057 | 0.000 | [-0.315, -0.092] |
| MVS 🡪 C | 0.060 | 0.060 | 0.050 | 0.226 | [-0.037, 0.158] |
| Condition 🡪 C | -0.146 | -0.146 | 0.106 | 0.167 | [-0.353, 0.061] |
| **Indirect effects:** |  |  |  |  |  |
| MVS🡪 Achievement 🡪 RS | -0.084 | -0.084 | 0.020 | 0.000 | [-0.123, -0.046] |
| MVS 🡪 Warmth and Reliability 🡪 RS | 0.003 | 0.003 | 0.012 | 0.785 | [-0.020, 0.027] |
| MVS 🡪 Emotional Expression 🡪 RS | 0.015 | 0.015 | 0.008 | 0.062 | [-0.001, 0.031] |
| MVS 🡪 Independence and Openness 🡪 RS | -0.006 | -0.006 | 0.007 | 0.362 | [-0.019, 0.007] |
| MVS 🡪 Positive Image 🡪 RS | 0.022 | 0.022 | 0.011 | 0.038 | [0.001, 0.043] |
| MVS 🡪 Achievement 🡪 C | 0.060 | 0.060 | 0.018 | 0.001 | [0.025, 0.095] |
| MVS 🡪 Warmth and Reliability 🡪 C | -0.002 | -0.002 | 0.007 | 0.785 | [-0.015, 0.011] |
| MVS 🡪 Emotional Expression 🡪 C | -0.003 | -0.003 | 0.005 | 0.635 | [-0.012, 0.005] |
| MVS 🡪 Independence and Openness 🡪 C | 0.009 | 0.009 | 0.006 | 0.131 | [-0.002, 0.021] |
| MVS 🡪 Positive Image 🡪 C | -0.012 | -0.012 | 0.006 | 0.040 | [-0.024, -0.001] |

## Figure A.

*Mediation model tested in Study 1 (N = 394).*

.15 (.24)**

.15 (.01)**

.17 (.07)**

Materialism

Interpersonal Conflict

Indirect effect: Materialism 🡪 Perceived discrepancies 🡪 Conflict: .02 (.01), 95% CI [.00, .06].

Total effect: Materialism 🡪 Conflict: .19 (.06), 95% CI [.12, .38].

R^2^ of Interpersonal Conflict = .057; R^2^ of Perceived discrepancies = .022.

Discrepancies between the actual

and ideal concept of

a significant other

*Note:* Significance levels: **p* < 0.05 level; ***p* < 0.01 (2-tailed).

## Figure B.

*Mediation model tested in Study 2 (N = 519).*

.10 (.16)*

.20 (.01)**

.06 (.05) *ns*

Materialism

Discrepancies between the actual

and ideal concept of

a significant other

Interpersonal Conflict

Indirect effect: Materialism 🡪 Perceived discrepancies 🡪 Conflict: .02 (.01), 95% CI [.00, .05].

Total effect: Materialism 🡪 Conflict: .04 (.05), 95% CI [-.01, .19].

R^2^ of Interpersonal Conflict = .051; R^2^ of Perceived discrepancies = .015.

*Note:* Significance levels: **p* < 0.05 level; ***p* < 0.01 (2-tailed).
